# Prevalence of pressure ulcers among the elderly living in long-stay institutions in São Paulo

**DOI:** 10.1590/S1516-31802009000400006

**Published:** 2009-12-07

**Authors:** Julieta Maria Ferreira Chacon, Leila Blanes, Bernardo Hochman, Lydia Masako Ferreira

**Affiliations:** 1 RN. Graduate student in the Master’s program on Plastic Surgery, Universidade Federal de São Paulo (Unifesp), São Paulo, Brazil.; 2 RN, PhD. Instructor and coordinator of the Wound Care Team at the Division of Plastic Surgery, Universidade Federal de São Paulo (Unifesp), São Paulo, Brazil.; 3 MD, PhD. Affiliate professor in the Division of Plastic Surgery, Universidade Federal de São Paulo (Unifesp), São Paulo, Brazil.; 4 MD, PhD. Full professor in the Division of Plastic Surgery and Coordinator of the Graduate Program on Plastic Surgery, Universidade Federal de São Paulo (Unifesp), São Paulo, Brazil.

**Keywords:** Prevalence, Pressure ulcer, Nursing homes, Health services for the aged, Nursing care, Prevalência, Úlcera de pressão, Casas de saúde, Serviços de saúde para idosos, Cuidados de enfermagem

## Abstract

**CONTEXT AND OBJECTIVE:**

The prevalence of pressure ulcers varies according to geographic region and population group, such as the institutionalized elderly. The aim of this study was to identify the prevalence of pressure ulcers among elderly people living in long-stay institutions.

**DESIGN AND SETTING:**

Cross-sectional study in six long-stay institutions for the elderly in São Paulo, Brazil.

**METHODS:**

Demographic and clinical data were collected in six long-stay institutions on two visits to each institution between May and August 2007, during which all elderly patients with pressure ulcers were evaluated. The Braden scale was used to identify the risk of developing pressure ulcers and the National Pressure Ulcer Advisory Panel (NPUAP) stages for classifying the pressure ulcers. Statistical analysis was performed using the chi-square test, Student’s t-test and Fisher’s exact test.

**RESULTS:**

There was no significant difference in the results between visits. The population was 181 elderly people in May and 184 in August: 23 had pressure ulcers in May (prevalence of 12.7%) and 17 in August (prevalence of 9.2%). The mean age at the two times was 84 years, and the average length of stay was 32 months. Pressure ulcers were found mainly in the sacral region (mean, 71.5%), and most commonly in stage II (mean, 41%).

**CONCLUSION:**

The prevalence of pressure ulcers was 10.95%. These data provide background information that may aid in developing protocols for applying best practices for prevention and treatment of pressure ulcers, consequently reducing the prevalence.

## RESUMO

**CONTEXTO E OBJETIVO:**

A prevalência de úlcera por pressão (UP) varia de acordo com a região geográfica e grupo populacional, como idosos institucionalizados. O objetivo foi identificar a prevalência de UP em instituições de longa permanência para idosos (ILPI).

**TIPO DE ESTUDO E LOCAL:**

Estudo transversal em seis ILPI em São Paulo.

**MÉTODOS:**

A coleta de dados demográficos e clínicos foi realizada em seis ILPI durante duas visitas a cada instituição no período de maio a agosto de 2007, sendo avaliados todos os idosos com UP. Foi utilizada a Escala de Braden para identificar o risco de desenvolver UP e o National Pressure Ulcer Advisory Panel (NPUAP) para a classificação das UP. Foram realizados os testes: Exato de Fisher, Q-Quadrado e t-Student para análise estatística.

**RESULTADOS:**

Não houve significância estatística nos resultados encontrados. A população era de 181 idosos em maio e 184 em agosto, sendo 23 idosos com UP (prevalência de 12,7%) e 17 idosos com UP (prevalência de 9,2%) respectivamente. A média de idade nos dois períodos foi de 84 anos, e 32 meses de permanência nas Instituições. A região sacral foi a mais frequente com média de 71,5% e as úlceras no estágio II com média de 41%.

**CONCLUSÃO:**

A prevalência de UP foi de 10,95%. Estes dados fornecem informações de base que podem auxiliar no desenvolvimento de protocolos para a aplicação de melhores práticas na prevenção e tratamento de úlceras de pressão, consequentemente reduzindo a prevalência.

## INTRODUCTION

The Brazilian population has been undergoing a rapid aging process since the beginning of the 1960s, when the decline in the fertility rate began to alter the age structure, thereby narrowing the base of the population pyramid. As a result, after 35 years, society faces a demand for social and medical services for the elderly that was once found only in industrialized countries. The national priorities are to reduce child mortality and control transmittable diseases and, thus, effective strategies for prevention and treatment of chronic degenerative diseases and their complications are still to be implemented. Within a context of major social and regional inequalities, the elderly do not receive adequate support from public healthcare services and social security. They therefore accumulate the sequelae of previous chronic diseases, develop disabilities and lose autonomy and quality of life.^1^^,^^2^

It has been observed that long-stay institutions have difficulties in maintaining the health of their elderly residents. These patients usually show declines in biological functions and they develop diseases that lead to a large variety of chronic conditions, thereby causing them to become debilitated and vulnerable.^3^ This is particularly the case with regard to pressure ulcers. These have been attracting more attention from healthcare professionals, health services and the general population, since their prevalence and incidence have increased in certain at-risk populations, despite preventive efforts and technological advances in treatment.^4^^,^^5^ These types of wounds are a cause for concern, because they are associated with prolonged hospital stay and are a common complication, especially among the institutionalized elderly.^6^ A study performed in the United States reported greater morbidity and mortality due to chronic diseases among patients living in long-stay institutions.^7^

There have been a number of discussions on causality, physiological pathogenesis and responsibility for prevention of pressure ulcers among high-risk patients, such as those with acute and chronic conditions and those receiving palliative care. Elderly individuals, particularly those living in long-stay institutions, are also at high risk.^3^ Among the major risk factors that contribute towards the development of pressure ulcers, advanced age, nutritional deficits, immobility, friction, diabetes and excessive moisture can be cited. Pressure ulcers are found mainly in the sacral, calcaneal, ischial and trochanteric regions.^3^^,^^8^^,^^9^^,^^10^^,^^11^^,^^12^

Among the elderly, debilitated sensory perception and decreased immune response may also be responsible for reductions in the inflammatory response to irritating factors. This would lead to longer exposure to these factors before a response such as hyperemia is triggered, thus delaying early intervention and healing. Greater numbers of comorbidities, the presence of chronic diseases (independent of whether individuals are aware of having them) and sedation episodes due to acute periods of illness may also lead to an increase in tissue trauma, thereby contributing to the formation of pressure ulcers.^3^^,^^11^

According to the National Pressure Ulcer Advisory Panel (NPUAP),^13^ the prevalence of pressure ulcers varies between countries, patient populations, institutions and study methodologies. There are limitations and difficulties in comparing data sets from different studies.^4^

Physical, social, psychological, familial and affective changes, as well as risk factors, contribute towards making elderly people living in long-stay institutions more susceptible to the development of pressure ulcers.^8^ Background knowledge regarding the prevalence of pressure ulcers in this population is important for developing strategies for their prevention and early treatment.

## OBJECTIVE

The aim of this study was to identify the prevalence of pressure ulcers among elderly people living in long-stay institutions in the western zone of São Paulo.

## METHODS

A list of all long-stay institutions for the elderly (LSIEs) in the western zone of São Paulo was provided by the agency responsible for the inspection of healthcare institutions in the city of São Paulo (COVISA). The western zone of São Paulo is an administrative region that comprises the districts of Lapa, Pinheiros and Butantã, and has a population of 888,623 (2000 census). The list comprised thirteen institutions, but four were excluded because they were not characterized as homes for the elderly, since individuals younger than 60 years were also admitted to these facilities. Another three institutions were excluded because research authorization was denied.

Thus, the overall population for the present study was the population living in six LSIEs in the western zone of São Paulo in May-August 2007. Our initial sample (n = 375) consisted of all of the patients living in the six LSIEs. Ten of these patients were excluded because consent was denied by relatives. Given this sample size, the study had a statistical power of 89% to detect an absolute difference of 3% with a 95% confidence interval, assuming a prevalence of pressure ulcers of 18.5% in LSIEs in Brazil.^14^^,^^15^

For the purpose of this study, the LSIEs were identified by the letters A, B, C, D, E and F in order to maintain confidentiality.

The study sample consisted of 365 patients aged 60 years and over who were living in an LSIE and agreed to participate in the study. The patients were distributed as follows: institution A, 35; institution B, 45; institution C, 85; institution D, 108; institution E, 42; and institution F, 50 patients.

This was a cross-sectional prevalence study that was conducted by counting the number of individuals with pressure ulcers in an elderly population over a given period of time. It comprised patients who were admitted with pressure ulcers and also those who developed pressure ulcers after admission to the institutions. The total number of elderly patients in residence at each institution was recorded on the data collection day.

This study was based on data collected during single-day visits to each institution, on two randomly-chosen days (one in May and another in August 2007), during which the monthly prevalence and the total mean prevalence were calculated for each LSIE.

Data were collected using an assessment instrument for identifying the characteristics of patients with pressure ulcers. All assessments were made by the first author. The patients’ skin was carefully inspected and all pressure ulcers were taken into account: even stage I pressure ulcers, which sometimes are overlooked by healthcare professionals because the skin is still intact. Upon detection, a thorough examination of the pressure ulcers was performed, and they were classified and recorded according to their stage and location (i.e. sacral, trochanteric, ischial or calcaneal regions, among others). Demographic and clinical data relating to the patients with pressure ulcers were collected from the patients’ records and through interviews. The pressure ulcers classification was in accordance with the NPUAP stages,^13^ and the pressure ulcer risk was assessed using the Braden scale.^16^ The Braden scale consists of six subscales: sensory perception, activity, mobility, moisture, nutrition, and friction and shear. Each subscale is rated from 1 to 4, except friction and shear, which is rated from 1 to 3. The sum of the ratings gives a total risk score (possible range: 6 to 23), with lower scores indicating higher risk. A cutoff score of 18 or less indicates increased risk for pressure-ulcer development.^4^^,^^16^

All evaluations were performed after written informed consent was obtained from the patients or their representatives. The study was approved by the Research Ethics Committee of the Universidade Federal de São Paulo (Unifesp), under process number CEP 1596/06.

Statistical analysis was performed using Pearson’s chi-square test, Student’s t-test and Fisher’s exact test. All statistical tests were carried out at a significance level of 5%.

## RESULTS

The study sample consisted of 365 elderly patients from six LSIEs located in the western zone of São Paulo. The demographic and clinical data collected from patient records and through interviews are listed in Table 1.

Each institution was evaluated separately during a one-day visit in May 2007 (first visit) and a one-day visit in August 2007 (second visit). Any pressure ulcers detected were thoroughly examined and evaluated according to their stage and location. There were no significant differences in the results between the visits.

**Figure 1.**
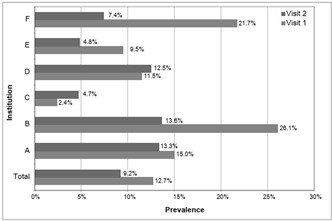
Prevalence of pressure ulcers in long-stay institutions for the elderly at each visit.

In May, 23 of the 181 elderly residents had pressure ulcers, i.e. a prevalence of 12.7% (standard error, SE = 2.48%), while in August, 17 of the 184 elderly residents (of which nine were new cases) had pressure ulcers, i.e. a prevalence of 9.2% (SE = 2.13%). The mean prevalence between the two visits was 10.95%. Figure 1 shows the prevalence of pressure ulcers in the six LSIEs (A-F) at each visit.

The patients with pressure ulcers were aged between 65 and 100 years in May (mean, 83.6 years), and between 67 and 97 years (mean, 83.8 years) in August.

In May, the length of stay among the patients with pressure ulcers ranged from one to 120 months (mean, 31.5 months), and in August, it ranged from one to 132 months (mean, 32.8 months).

Among the patients with pressure ulcers, urinary incontinence was present in nine individuals in May and in seven in August, while fecal incontinence was present in seven in May and in four in August.

The conditions presented by the patients at admission were: hypertension (47.5%), cardiovascular and respiratory diseases (37.5%), neurological disorders (27.5%), senile dementia (22.5%), Alzheimer’s disease (15%), diabetes mellitus (12.5%) and neoplasia (2.5%).

Among the medications most used by the patients were vitamins, antacids, cerebral vasodilators, ferrous sulfate and platelet antiaggregants (90%), followed by antihypertensive medications (45%), anxiolytics and antidepressants (27.5%), hypoglycemics (12.5%), cardionics (10.5%), diuretics (7.5%) and analgesics (2.6%).

At the first visit, 18 of the 23 patients with pressure ulcers presented high risk of pressure ulcers according to the Braden scale, while at the second visit, nine of the 17 patients with pressure ulcers presented high risk. There was no significant difference in the distribution of risk levels for pressure ulcers between the visits.

Most of the patients with pressure ulcers (14 of the 23 patients in May, and 10 of the 17 patients in August) scored two (probably inadequate) on the Braden nutrition subscale.

With regard to the sensory perception subscale, 10 of the 23 patients with pressure ulcers in May scored three (slightly limited), while 10 of the 17 patients with pressure ulcers in August scored two (very limited).

According to the moisture subscale, 11 of the 23 patients with pressure ulcers in May had a score of two (very moist), while 11 of the 17 patients with pressure ulcers in August scored two.

Most of the patients with pressure ulcers (18 out of 23 patients) had a score of one on the activity, mobility and friction and shear subscales at the first visit. These correspond to bedridden, completely immobile and problem conditions, respectively.

**Table 1.** Demographic and clinical characteristics of the elderly individuals with pressure ulcers for each month of data collection

Characteristics	May		August		P value
	n	%	n	%	
Sex					0.144
Male	1	4.3	4	23.5	
Female	22	95.7	13	76.5	
Skin color					
White	21	91.3	17	100	0.499
Nonwhite	2	8.7	0	0	
Marital status					
Single	4	17.4	2	11.8	0.841
Married	1	4.3	1	5.9	
Widowed	18	78.3	14	82.4	
Religion					
Catholic	22	95.7	17	100	1.00
Protestant	1	4.3	0	0	

## DISCUSSION

Brazil has a population of 14.5 million individuals aged over 60 years, which corresponds to 8.6% of the total Brazilian population. Over the next 20 years, the Brazilian elderly population is predicted to reach approximately 30 million individuals, which will represent almost 13% of the total Brazilian population by the end of this period.^1^^,^^2^

A study conducted in 2000 revealed the characteristics of individuals aged 60 years or over living in the city of São Paulo, and showed who they were, how they lived and how healthy they were. This study reported that the elderly people had a mean age of 69 years and a mean schooling level of 3.4 years, and also that 8.9 million (62.4%) of this population were responsible for their own homes. The elderly population comprises 9.3% of the population of São Paulo, the state capital, according to the Brazilian Institute for Geography and Statistics (Instituto Brasileiro de Geografia e Estatística, IBGE).^1^^,^^2^

The prevalence of pressure ulcers in the six LSIEs located in the western zone of São Paulo was consistent with other studies and results in the literature.^12^^,^^17^^,^^18^^,^^19^ Studies in other countries have reported prevalences of pressure ulcers in LSIEs of between 7% and 23%.^4^^,^^7^^,^^12^^,^^17^ In Brazil, only a few studies on the incidence and prevalence of pressure ulcers have been conducted in LSIEs, including the study by Souza and Santos,^3^ who reported an incidence of 39.4% in a study performed in four LSIEs in southern Minas Gerais.

Pressure ulcers are a common problem in various healthcare settings, and are frequently found in acutely or chronically ill patients living in LSIEs for long periods of time.^3^^,^^8^

Demographic and clinical data were collected in six long-stay institutions on two visits to each institution. Most of the patients with pressure ulcers were females (35 out of 40 patients, 87.5%). There were 22 women (95.7%) and one man (4.3%) with pressure ulcers on the first visit, and 13 women (76.5%), of which five were new cases, and four men (23.5%, all new cases) with pressure ulcers on the second visit. This result is consistent with those reported by Brandeis et al.^7^ (69%) and Souza and Santos^3^ (62.8%).

The patients included in this study were aged between 65 and 100 years (mean, 84 years; median, 84-86 years). There was no significant correlation between advanced age and greater incidence of pressure ulcers, which is consistent with the findings of Souza and Santos^3^, although other authors have reported contrary results.^20^^,^^21^

It is known that the length of hospital stay may be extended by the presence of pressure ulcers, which require specific targeted interventions. The results from one study revealed that the incidence of pressure ulcers was 7.7% among patients who had been bedridden and chairbound for more than three weeks, and 50% among patients older than 70 years of age. The same study also showed that pressure ulcers were in fourth position among the leading causes of death among elderly patients receiving home care.^7^ In the present study, 325 elderly patients (89%) did not have pressure ulcers. At admission, only eight patients (2%) had pressure ulcers: three of them (0.8%) came from their own homes, four (1%) from hospitals and one (0.2%) from another LSIE. There was no significant correlation between the incidence of pressure ulcers and length of stay in the LSIE, which ranged from one to 132 months of stay (mean, 32 months).

Diseases relating to neuromotor and skeletal muscle disorders impair the quality of life, self-care and functional capacity of elderly people. Consequently, such diseases interfering with elderly people’s nutrition and levels of sensory perception, mobility, activity and skin moisture, which are the classic risk factors for pressure ulcers.^16^

Another important factor in the development of pressure ulcers is excessive exposure of the skin to moisture due to urinary and fecal incontinence and perspiration. This moisture macerates and weakens the skin, which thus becomes more susceptible to injuries, especially those caused by friction and shear.^6^^,^^10^^,^^14^^,^^17^^,^^18^

Urinary incontinence and fecal incontinence are medical conditions that require careful handling, since prejudice, myths and taboos are involved. This makes diagnosis and treatment a difficult task, and leads to high prevalence of skin moisture, which is frequently the main reason why elderly people are admitted to LSIEs.^3^

Our results showed that 27.5% of the participants had urinary incontinence and 40% had fecal incontinence. Although there were no significant differences between the results from the participating institutions, these patients characterized a population at greater risk of pressure ulcers.

In this sample, there was no significant correlation between use or nonuse of medications and the development of pressure ulcers. It is known that continuous use of medication, although necessary, may contribute towards the development of pressure ulcers. Sedatives and analgesics, for instance, reduce the pain but affect mobility. On the other hand, antihypertensive agents may affect blood flow, thereby reducing tissue perfusion and making the tissue more susceptible to pressure.^5^^,^^6^^,^^11^

Pressure ulcers were found mainly in the sacral, trochanteric, ischial and calcaneal regions. Bedridden patients who remain in a supine position are subjected to higher pressure on the sacral region. Patients who remain in lateral positions or remain sitting on chairs with limited mobility may develop pressure ulcers in these pressure areas.^4^^,^^5^^,^^6^^,^^14^^,^^16^^,^^20^^,^^22^

With regard to pressure ulcer staging, 26 of the 51 ulcers (51%) that were found on examination were stage II, which is in agreement with findings from other authors^3^^,^^4^^,^^7^^,^^12^ who reported higher incidence of pressure ulcers at stages II to IV. It is important to mention that the number of stage I pressure ulcers (pre-ulcers) may be an underestimate because they are hard to detect. Sometimes it is hard for a nurse to distinguish between a reactive and a non-reactive erythema, which may evolve to advanced stages if the causative stimulus is not removed.^5^^,^^6^

The Braden scale scores ranged from 7 to 19, with means of 10.35 and 10.76 for the first and second visits, respectively. There was no significant difference between the visits. According to the Braden scale, 67.5% of the patients were at high risk of pressure ulcer development.^16^^,^^18^

In this study, more than 50% of the patients had low scores on the sensory perception, mobility, nutrition and friction and shear subscales. Decreased sensory perception associated with low mobility result in lack of response to pressure among elderly individuals, who become more susceptible to friction and shear, and to the development of pressure ulcers. These patients are considered to be at high risk of pressure ulcers according to the Braden scale.^14^^,^^16^^,^^20^^,^^23^

The aging of the world’s population is a problem, and better knowledge of this dynamic and irreversible process is needed in order to address the resulting increase in vulnerability and fragility of this population. Pressure ulcers are an important cause of health loss that aggravate other health problems and inhibiting their cure, thereby increasing suffering, morbidity and nursing care time.^24^^,^^25^

The aim of the present study was to draw attention to the importance of developing protocols and using risk assessment scales for pressure ulcers. This study also highlights the need for a multidisciplinary team to help elderly people maintain their independence and autonomy for as long as possible, minimize their suffering and reduce the social and economic impact.

## CONCLUSION

The prevalence of pressure ulcers among the elderly population living in long-stay institutions in the western zone of São Paulo was 10.95%. Our results provide background information that may be useful in designing best-practice protocols for prevention and treatment of pressure ulcers, thereby reducing their prevalence.
